# Nanomaterials and nanotechnology for the delivery of agrochemicals: strategies towards sustainable agriculture

**DOI:** 10.1186/s12951-021-01214-7

**Published:** 2022-01-04

**Authors:** Changcheng An, Changjiao Sun, Ningjun Li, Bingna Huang, Jiajun Jiang, Yue Shen, Chong Wang, Xiang Zhao, Bo Cui, Chunxin Wang, Xingye Li, Shenshan Zhan, Fei Gao, Zhanghua Zeng, Haixin Cui, Yan Wang

**Affiliations:** Institute of Environment and Sustainable Development in Agriculture, Chinese Academy of Agricultural Sciences, Beijing, 100081 China

**Keywords:** Nanomaterials, Agrochemicals, Nanotechnology, Nanoformulations, Sustainable agriculture

## Abstract

Nanomaterials (NMs) have received considerable attention in the field of agrochemicals due to their special properties, such as small particle size, surface structure, solubility and chemical composition. The application of NMs and nanotechnology in agrochemicals dramatically overcomes the defects of conventional agrochemicals, including low bioavailability, easy photolysis, and organic solvent pollution, etc. In this review, we describe advances in the application of NMs in chemical pesticides and fertilizers, which are the two earliest and most researched areas of NMs in agrochemicals. Besides, this article concerns with the new applications of NMs in other agrochemicals, such as bio-pesticides, nucleic acid pesticides, plant growth regulators (PGRs), and pheromone. We also discuss challenges and the industrialization trend of NMs in the field of agrochemicals. Constructing nano-agrochemical delivery system via NMs and nanotechnology facilitates the improvement of the stability and dispersion of active ingredients, promotes the precise delivery of agrochemicals, reduces residual pollution and decreases labor cost in different application scenarios, which is potential to maintain the sustainability of agricultural systems and improve food security by increasing the efficacy of agricultural inputs.

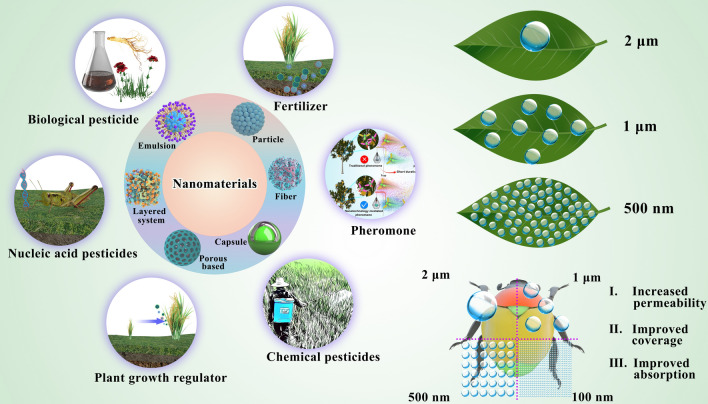

## Introduction

Particles with nanometers sizes in at least one dimension are commonly known as nanomaterials (NMs) [1, 2]. Due to their small size, NMs are used in many fields, such as life sciences, aerospace, electronics, chemical production, and agriculture. Many inorganic nanoparticles are employed as NMs, for example, carbon nanotubes are usually used as carriers; Iron nanoparticles are widely used due to their magnetism; Silica nanoparticles have attracted research attention for their abundant pore structure and large specific surface area. Other studies have focused on copper, gold, or silver nanoparticles [3]. Polymers and liposomes are renewable, biodegradable, and environmentally friendly, usually used as organic carriers to encapsulate pesticides [4, 5]. These materials are also called nanostructured materials (NSMs) or engineered nanomaterials (ENMs), and most applications are concentrated on efficiency and productivity enhancement.

**Fig. 1:**
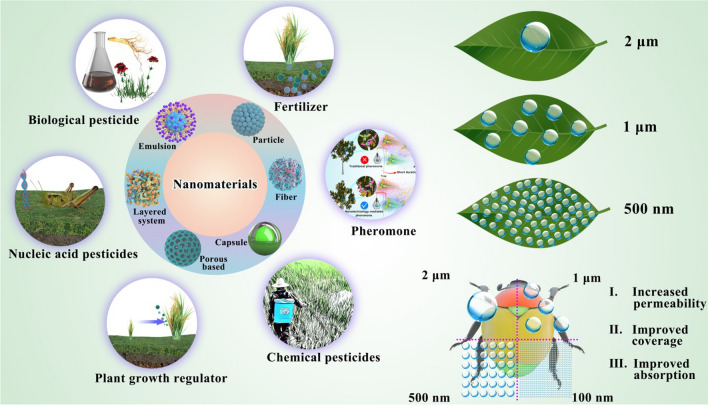
Nanomaterials loaded with various agrochemicals. Multiple release modes of active ingredients by modulating the structure of the loading system (Particle, Fiber, Layered system, Porous based, Capsule, Emulsion). The small size effect and large specific surface area of nanoparticles can adhere to the target as much as possible to improve the bioavailability of active ingredients

Roco et al. were the first to note that NMs can be used for drug delivery [6]. A National Planning Workshop of America proposed the use of NMs in agriculture, mainly for rapid detection of pesticide residues and food packaging [7]. Subsequent research on the application of NMs has involved various aspects of agrochemicals such as pesticide formulations [8, 9], pesticide residues analysis [10, 11], fertilizers [12, 13], plant growth regulators (PGRs) [14], and development of bio-pesticides [15, 16], including nucleic acid pesticides and pheromone. The application of NMs in agrochemicals is shown in Fig. 1. In 2019, IUPAC selected ten chemical technologies most likely to impact human society in the future [17], and nanopesticides were ranked first for their potential low impact on the environment and human health. Benefits such as increased penetration [18], coverage [19] and uptake of active ingredients (AIs) [20] on the target in the presence of NMs will also occur for all agrochemicals with nanotechnology applications in agriculture [21]. Nano-agrochemical delivery system via NMs have great potential for improving the efficacy of agricultural inputs, alleviating environmental pollution, and saving labor costs, which contributes to the maintenance of the sustainability of agricultural systems and the improvement of food security. NMs will have prospective future in agriculture [22].

Although NMs have promising applications in agrochemical delivery [23, 24], academics have also raised some concerns [25], mainly in terms of the unknown health risks for human beings and regulatory difficulties brought by the small size effect of NMs. The complexity and cost of manufacturing technology also limit the large-scale use of NMs in agriculture [26]. In this paper, we illustrate the recent advances and challenges of NMs applied as plant protection agrochemicals.

## Nanomaterials for pesticide and fertilizer applications

Pesticides and fertilizers are the most widely used agrochemicals in agricultural production [27], and NMs were the earliest to be applied to these two agrochemicals which are also the most researched and reported. Constructing pesticides and fertilizers delivery system through different strategies could improve their utilization and reduce the run-off to environment, which contributes to alleviating environmental pollution and negative impact on the non-target organism caused by excessive use of agricultural inputs.

### Nanomaterials for the establishment of pesticide formulations

Pesticides play a crucial role in defending against biological disasters and promoting crop productivity. Most AIs of pesticides are lipid-soluble [28]. Preparing AIs into liquid formulations requires numerous organic solvents; emulsifiers and other additives are also essential. These requirements can trigger overuse of organic solvents, posing a threat to the environment and human health. Solid formulations need solid emulsifiers and many inert materials as fillers, and they are widely used due to their easy storage and transportation. However, dust pollution is caused in the process of production and application, because of lacking effective protection measures, which is prone to harming non-target organisms as well. Wettable powder (WP) and emulsifiable concentrate (EC) are main pesticide formulations in conventional pesticide products [29, 30], but their actual AIs utilization of biological target uptake is less than 0.1% after dust drift and rainwater leaching [31].

**Fig. 2:**
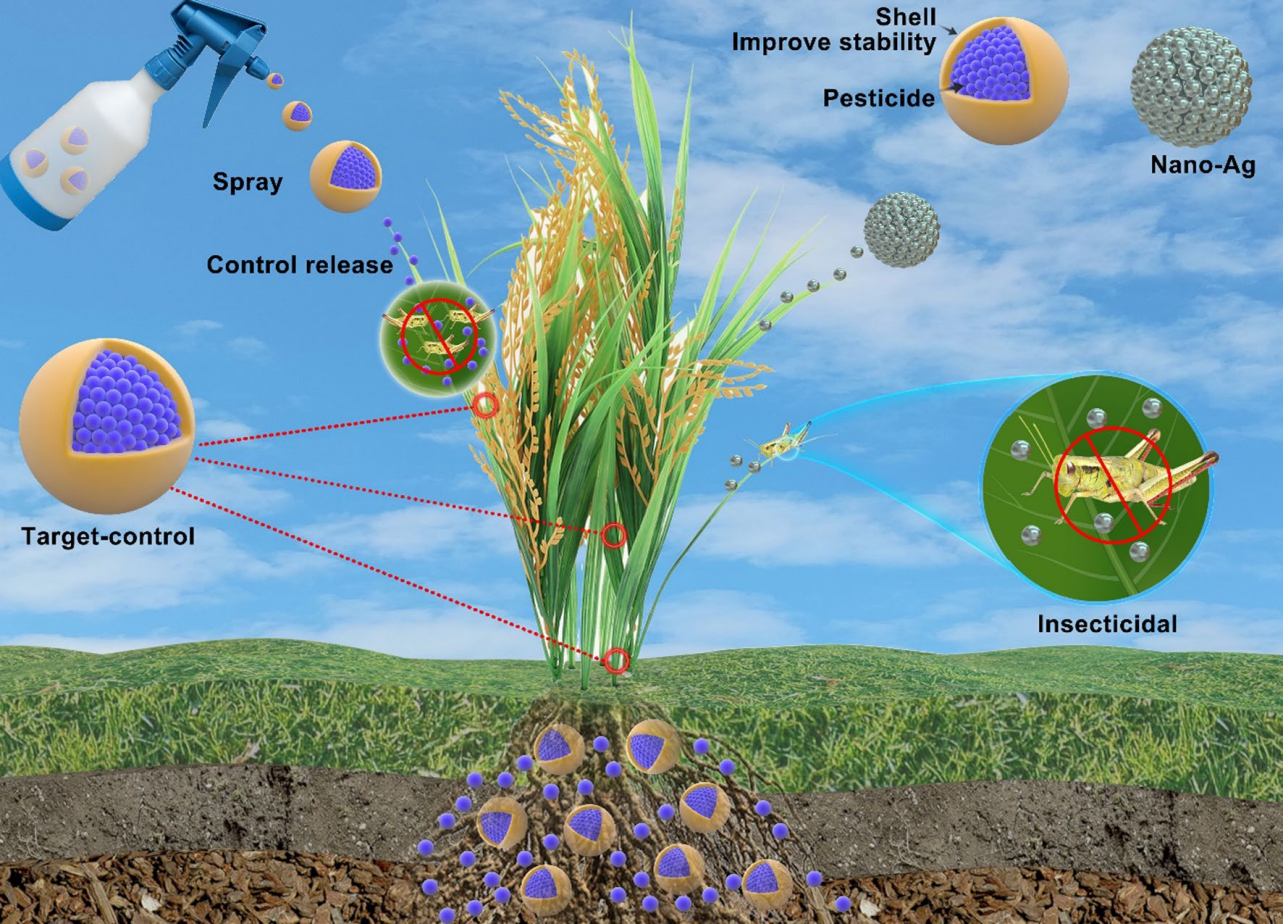
Nanomaterials loaded with pesticides. NMs loaded with pesticides can reduce the degradation of AIs caused by environmental influences, achieve target-controlled release of AIs by the properties of nanomaterials, thus prolonging the shelf life of pesticides; Some NMs have their own insecticidal and fungicidal activities; When applied to soil, NMs help pesticides to be fixed in the soil for plant root absorption in order to reduce the loss of pesticides by leaching

NMs used in pesticide production mainly participate in establishing nanopesticide formulation systems to improve the bioavailability of AIs (Fig. 1), detailed reasons as follows (Fig. 2).

*NMs improve the stability of AIs.* AIs that easily undergo photolysis and are easily decomposed can be stabilized under the protection of NMs. Avermectin (AV) is an excellent bio-pesticide that has been widely used, but it is sensitive to ultraviolet (UV) light and has a very short half-life (6 h) [32]. Zhu et al. grafted AV onto the long chain of poly (ethylene glycol)-carboxymethyl cellulose (PEG-CMC), and the obtained nanoparticles improved the stability of AV significantly over the control, and the degradation rate of AV was less than 50% after 72 h of illumination [33]. Another approach is using NMs as a protective shell for AIs. Kaziem et al. encapsulated AV in hollow mesoporous silica, then anchored cyclodextrin in the outermost layer of the particles [34]. AV was well protected, as less than 9% of AV in these nanoparticles was photolyzed after 24 h UV radiation. Similar to AV, some unstable pesticides such as phoxim, fosthiazate, and quinalphos would likely also be protected by using NMs. Other NMs including lignin and carbon-based materials can also be used as a shell material to protect AIs [35; 36].

*NMs can be used for targeted and controlled release of AIs at the optimum working concentration.* This prevents waste of AIs and accidental harm to non-targets, and reduces the risk of resistance caused by concentrations of AIs that are too high or too low [37]. Generally, these materials are nanoscale polymers generated by reactions under specific conditions, such as urea–formaldehyde resin [38], polyurethane [39] as well as polyurea [40]. Xiang et al. prepared a magnetic nanocarrier consisting of diatomite and Fe_3_O_4_ by in situ deposition. In this formulation, both herbicide (glyphosate) and insecticide (cypermethrin) were wrapped, then coated with chitosan, which could be dissolved to control the release of the pesticide under acidic conditions. The magnetic properties of Fe_3_O_4_ help separate nanocarriers from soil and water, facilitating recycling of the materials [41].

Shan et al. used sulfite ions as ring-opening reagents to convert epoxy groups into sulfonic acid groups, and introduced sulfonic acid groups on mesoporous silica nanoparticles (MSNs) by post-grafting to easily fabricate negatively charged decorated MSN-SO_3_. Diquat dibromide (DQ), the positively charged herbicide was effectively loaded by electrostatic interaction in these negatively charged MSN-SO_3_ nanoparticles. The loading rate increased to 12.73% for MSN-SO_3_ compared to unmodified MSN alone as a carrier (5.31%). The release of DQ from DQ@MSN-SO_3_ nanoparticles was dependent on pH and ionic strength, which were mainly determined by electrostatic interactions. Bioactivity studies showed that DQ@MSN-SO_3_ nanoparticles exhibited good herbicidal activity in controlling *Datura stramonium* L. compared to the control [42]. Xiang et al. obtained a pH-reacting controlled-release formulation using attapulgite-based hydrogel. Chlorpyrifos (CPF) was adsorbed to the nano-mesh structure polydopamine modified attapulgite (PA) through hydrogen bonding and electrostatic interaction to obtain CPF-PA. CPF-PA and calcium alginate (CA) subsequently formed porous hydrogel spheres of CPF-PA-CA via a cross-linked reaction, where PA was the skeleton. Under alkaline conditions, the outer layer of material disintegrated and the AIs were released. With the protection of the material, the utilization rate of the AIs was improved and the duration of pesticide activity was prolonged [43].

NMs could decrease the toxicity of pesticides to the non-targets by taking advantage of the isolation effect of the material on the AIs and the organism, which are helpful for enlarging the applicable area of pesticides. Pyraclostrobin has shown excellent control of rice blast disease. This chemical triggers physiological changes in crops, improving their tolerance to the disease and thus significantly increasing yields [44]. However, this compound is highly toxic to aquatic organisms, limiting its application to rice crops [45]. Based on this situation, Li et al. prepared and obtained pyraclostrobin-loaded polyurea nano microcapsules through an interfacial polymerization reaction between isocyanates and amines. The particle size distribution ranged from 815 to 867 nm, forming nearly spherical shapes. This method will reduce pesticide toxicity to aquatic species by coating pyraclostrobin, contributing to improved environmental safety and allowing pyraclostrobin to be applied to rice [46]. Chi et al. developed a core–shell structured magnetic-responsive controlled-release herbicide by a nanocomposite including palygorskite, ferroferrie oxide, glyphosate and amino silicon oil. The release of glyphosate could be accelerated under a magnetic field, and the incomplete coating of amino silicon oil could effectively prevent the herbicide from being completely exposed to the environment and reduce the loss. Biosafety experiment to zebrafish showed that this delivery system had better biosafety than glyphosate [47].

*NMs could be directly used as nanopesticides due to their antibacterial or insecticidal properties.* NMs can easily penetrate cell membranes due to their nanostructure and small size, and the surface properties of small materials allow them to attach to cell organelles, influencing normal cell organ function and directly or indirectly generating abnormal reactive oxygen species (ROS) [48]. In important physiological processes such as cell signaling, gene expression, and protein redox regulation, a normal ROS level is essential [49]; however, high ROS levels in the above cellular processes cause anomalies and interrupt normal functioning, resulting in cell death [50].

Nanosilver, for example, has biotoxic properties. Bharani and Namasivayam synthesized nanosilver with good control effect against *Spodoptera litura* [51]. According to the research of Meng et al., the results of in vitro studies showed that silver nanoparticles enter cells through phagocytosis [52, 53] or by passive diffusion [54]. When nanosilver enters the cytoplasm, they appear in intracellular vesicles and can enter mitochondria, nuclei or other organelles [55]. The toxicity of nanosilver depends on particle size. Tang and Zheng summarized the research on the relationship between silver nanoparticles size and its antibacterial properties, and they concluded that the size of silver nanoparticles is a key factor in their antibacterial activity, silver nanoparticles with an average size of 15 nm showed better antibacterial activity against *E. coli* than the silver nanoparticles with an average size of 75 nm [56]. Shafie et al. [57] used five different concentrations (25, 50, 100, 150 and 200 mg/L) of silver nanoparticles as antiviral agents to reduce tomato spotted wilt virus (TSWV) infestation on *Ch. amaranticolo*r and potato plants. As a result, the different concentrations of silver nanoparticles significantly inhibited the number of local lesions produced by the leaves of *Ch. amaranticolor* inoculated with TSWV. The inhibition effect was significant (90.4%) after 24 h of spraying silver nanoparticles.

*NMs have small size and high specific surface area effect.* NMs as carriers increase the solubility of AIs while protecting them from volatilization and degradation [58]. Furthermore, the particle size of pesticide influences the utilization of pesticides [59]. Based on the small size effect and high specific surface area of NMs, more nanoparticles containing AIs of pesticide can be attached to the target per unit area, significantly increasing the area where AIs contact the target and increasing the bioavailability of the AIs [60]. Liu et al. used polylactic acid (PLA) to coat the Lambda–Cyhalothrin, and through the control of process parameters, various drug delivery systems with adjustable particle size between 0.68 μm and 4.6 μm were produced, and they found that the microencapsulated drug delivery systems had good water dispersion and obvious slow-release properties, and the 0.68 μm microcapsules had good biological activity and UV and thermal stability [61]. Wang et al. similarly used PLA to investigate the construction of different particle sizes of AV nano-delivery systems and evaluated their performance. The release rate of AV in the nano-delivery system could be effectively controlled by varying particle sizes, and the results showed that the biological activity was enhanced with decreasing particle size [62].

Generally speaking, the benefits of NMs in delivering Als are obvious, such as improving the stability, increasing utilization rate and bioactivity, and extending shelf life. Studies have shown that the release of Als from delivery systems could be regulated according to the properties of delivery materials and application scenarios. One case in point is pH-sensitive release. Li et al. constructed pH-sensitive thiamethoxam nanoparticles through free radical polymerization and found that the rupture of these nanoparticles in the alkaline intestinal environment of peach aphid could achieve the regulated release of Als [63]. Similar modes include enzyme-sensitive release [64], temperature-sensitive release [65], etc. Usually, establishing indoor release curves is the most commonly used method to determine the sustained-release model of Als, and the release experiments are usually carried out in water or a mixture of water and water-soluble organic solvents [66]. However, it is obvious that the release environment simulated in the laboratory is rather simple, while the real-world environment for release is complex and changeable, as pH, salinity, light, microorganism, and other factors may exert individual or interactive influences on the release of pesticides. Therefore, it is essential to examine the correlation between laboratory-based release model and real-world release model, so as to establish a more accurate and intelligent pesticide delivery system through laboratory studies. This, of course, is still challenging and further studies are needed.

### Nanomaterials for fertilizer applications

**Fig. 3:**
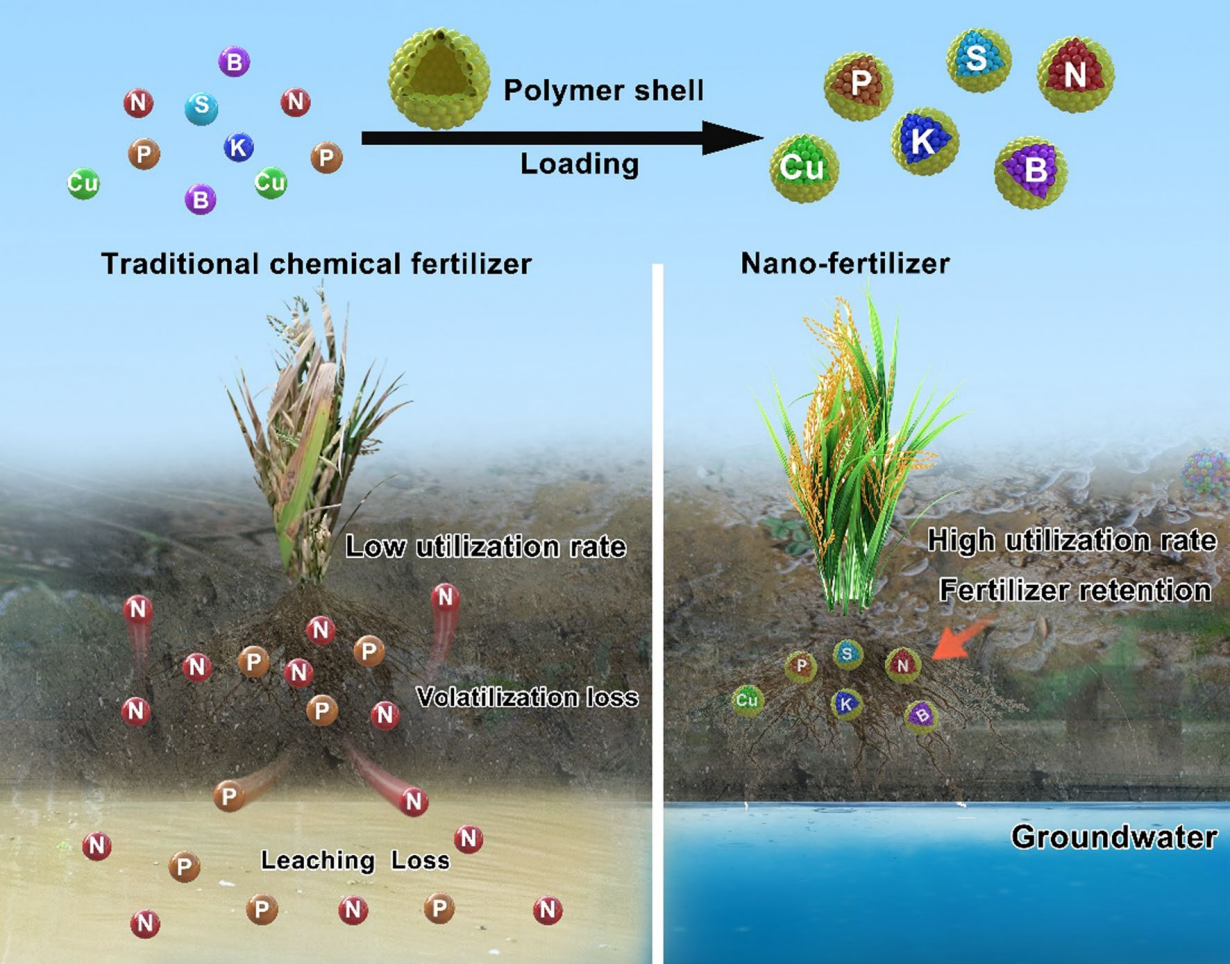
Nanomaterials loaded with fertilizers. Nanomaterial loading of fertilizer. Loading fertilizer with nanomaterials can achieve slow release of fertilizer and improve fertilizer utilization, thus reducing the environmental problems caused by fertilizer loss. In soil, fertilizers were fixed by NMs to facilitate plant roots absorption, reduce the leaching loss of fertilizer, and minimize the pollution of groundwater

Currently, chemical fertilizers are favored by farmers [67], because they are more effective and economical than other fertilizers. However, chemical fertilizers can be overused and even wasted, causing soil damage, food yield reduction, and environmental pollution (Fig. 3.). For example, eighty percent of plant demand for N-fertilizers is fulfilled by urea, which is extremely water-soluble and prone to loss [68]. If any N compound is applied to a paddy field, then liquidation, denitrification, volatilization, and runoff cause leaching. Leaching accounts for about 30% to 50% of total N loss, primarily as nitrate, denitrification, about 10–30% as N_2_, volatilization, and about 2–30% as ammonia [69]. Escaped nitrogen pollutes air and water systems, causing eutrophication. The eutrophication of water contributes to hypoxia and results in many environmental concerns, including mortality of marine animals and algal blooms, and poses a threat to drinking water supplies.

As shown in Table 1, NMs have been used in various fertilizers. They are fabricated as controllable and tunable fertilizers and applied to improve fertilizer usage. These formulations enhance nutrient absorption by optimizing soil nutrient management, helping to create a nutrient cycle in agriculture, relieving depletion of nutrient resources, and minimizing the nutrient disordered effect on crop output and the environment. NM-mediated nano-fertilizers can also be used in precision agriculture, with specialist polymers or methods, activated by signals secreted in the environment by plant roots [70]. NMs now are specifically applied as bio-fertilizer; trace element fertilizers such as Fe and Zn; major element fertilizers, including N, P, and K; medium element fertilizers, for example, Si, Ca, Mg, and S. NMs are also used in organic fertilizer. In this section, we present an overview of the application of NMs for each of the fertilizer types as above.

**Table 1:** Nanomaterials applied to fertilizer and their functions

Fertilizer category	Active Ingredients (AIs)	Material(s)	Function	Refs
Bio-fertilizer	growth promoting rhizobacteria (PGPR) & endophytic bacteria	graphite and silica composites	The nano formulation has wider inhibition zones, indicating that this delivery system affects the control mechanism	[71]
Trace element fertilizers	Iron (Fe_3_O_4_)	Iron oxide nanoparticles as well as EDTA-functionalized iron oxide nanoparticles	Improve in total chlorophyll, total carbohydrates, germination rate, and iron content of the plants and increase plant height, sugar content, and antioxidant enzymes of mulberry (Morus alba L.)	[73]
	Zinc (Zn)	Use fungi preparation of Nano – Zn	The application of Zn nano-fertilizer increased the yield of the crop at maturity by 37.7%	[74]
	Selenium (Se)	Nano – Se	Help minimize pesticide induced oxidative damage of tea	[75]
Major element fertilizers	Nitrogen (N)	Nano-Nitrogen Chelate	Increase the sugar content of sugarcane and reduce the leaching of N in the soil	[76]
	Phosphorus (P) and potassium (K) compound	zeolite	The potassium content in the soil was maintained at a higher level with the nano-potassium fertilizer	[77]
Medium element fertilizers	Calcium (Ca)	Nano – Ca_2_SO_4_	Nano calcium sulfate provides a larger surface area, higher solubility, and improved integration of fertilizer and soil, which further reduces soil phosphorus loss, and it is a fertilizer itself	[78]
Organic fertilizer	Vermicompost	Nano-vermicompost organic fertilizer	Beneficial effects on growth, oxidative stress parameters, antioxidants, nitrogen metabolism, osmotic pressure accumulation and mineral elements in tomato under drought stress	[79]

Djaya et al. developed a composite embedding process for graphite and silica composites to encapsulate the plant growth promoting rhizobacteria (PGPR) and endophytic bacteria to obtain a novel biocontrol delivery system (BDS) [71]. The efficacy of the BDS was evaluated by measuring the diameter of inhibition zone against *R. solanacearum* culture. The results showed that compared to control, inhibition zone of the treatment of *Lysinibacillus sp.* was up to 10.24 times wider, and treatment of *Bacillus sp.* was 12.6 times respectively, which indicated the ability of BDS to form synergistic effect and inducing antagonistic interactions.

Benzon et al. found that compared to conventional fertilizer use only, combining nano-fertilizer with conventional fertilizer significantly improved the agronomic parameters of rice [72]. The plant height, chlorophyll content, number of reproductive tillers, panicles and spikelets were increased by 3.6%, 2.72%, 9.10%, 9.10% and 15.42%, respectively. Panicle weight, total grain weight, total shoot dry weight and harvest index were also improved by 17.4%, 20.7%, 15.3%, and 2.9%, respectively. Furthermore, the authors determined that the performance of using only nano-fertilizer was not noticeable and suggested using nano-fertilizer in conjunction with conventional fertilizer to achieve good results.

Haydar et al. evaluated the biological effects of iron oxide nanoparticles as well as EDTA-functionalized iron oxide nanoparticles as a nano-micronutrient fertilizer in place of conventional iron fertilizers [73]. The effect of these fertilizers on agronomic trait parameters of mulberry (*Morus alba *L.) was estimated in a pot experiment. The application was carried out by both foliar spraying and soil application, and iron oxide nanoparticles and their EDTA functionalized forms were applied at two different concentrations (10 and 50 mg/kg soil). The results showed positive effects on the agronomic trait parameters of the treated plants. Iron oxide nanoparticles in the soil at a concentration of 10 mg/kg significantly improved the morphological characteristics of the plants, such as germination rate, leaf number (52.73% higher than the control), plant biomass (37.20% and 90.24% increase in shoot and root biomass, respectively, compared to the control), root properties (34% increase in root length), and also reduced the time to germination of the first leaves. The same treatments increased by 42% and 15%, respectively, compared to the control for chlorophyll and sugar content. The activity of antioxidant enzymes such as catalase, peroxidase and NADPH oxidase was also increased compared to the control. All results showed that iron oxide nanoparticles could replace the traditional iron fertilizer in the cultivation and propagation of mulberry crops.

Raliya et al. developed nano zinc fertilizer to increase the yield of pearl millet (*Pennisetum americanum*) [74]. In this study, the fungus *Rhizoctonia bataticola* TFR-6 (NCBI GenBank Accession number JQ675307) was cultivated in potato dextrose (PD) broth medium. The filtrate of fungus-free organism cells was isolated, which was used to prepare an aqueous zinc oxide solution with a concentration of 0.1 mM. The synthesis of nano zinc was performed by exposing the former zinc oxide solution to the fungal cell-free filtrate previously prepared for 62 h. The dynamic light scattering (DLS) results showed that the particle size of the synthesized nanoparticles ranged from 15 to 25 nm and had spherical with clear particle interfaces and crystalline structures as seen by TEM. Fertilization with the nano-fertilizer applied to 6-week-old pearl millet showed the following results: stem length (15.1%), root length (4.2%), root area (24.2%), chlorophyll content (24.4%), total soluble leaf protein (38.7%), plant dry biomass (12.5%), acid phosphatase (76.9%), alkaline phosphatase (61.7%), phytase (322.2%) and dehydrogenase (21%) activities were significantly higher than the control. The application of Zn nano-fertilizer increased the yield of the crop at maturity by 37.7%.

Li et al. found via field and indoor experiments that the long-term use of pesticides in tea gardens would cause the plants to accumulate excessive amounts of ROS, leading to oxidative damage in the plants and affecting the nutritional quality of tea [75]. In contrast, selenium nanoparticles activated the plant antioxidant system, resulting in significant increases in the content and activity of antioxidant enzymes in the ascorbic acid-glutathione cycle, which helped minimize pesticide induced oxidative damage. Biofortification with selenium significantly increased the accumulation of nutrients and functional substances such as proteins, carotenoids, tea polyphenols, and catechins in tea products. Metabolomic and transcriptomic studies have shown that selenium nanoparticles can induce theanine, glutamate, proline, and arginine production by regulating the glutamine-glutamate cycle. Nanoselenium exogenous supplementation promotes secondary metabolism in tea plants, which in turn increases the biosynthesis of total phenols and flavonoids in tea leaves. Nanoselenium promotes the uptake and conversion of selenium in the soil, leading to a significant increase in the production of selenium-rich teas with many key aroma components.

Alimohammadi et al. evaluated the effect of urea and Nano-Nitrogen Chelate (NNC) fertilizers on yield of sugarcane (*Saccharum Officinarum*) and nitrate leaching from soil [76]. Treatments included five different N contents of urea and NNC (0, 80, 112, 137 and 161 kg N ha^−1^). Results showed that the mean values of soil nitrate concentrations were 10.2 and 12.8 mg/kg for urea and NNC treatments, respectively, during sugarcane growth. The nitrate leaching level was high with urea fertilizer (699.0 mg l^−1^) and low with NNC fertilizer (183.0 mg l^−1^). This study also found that the application of NNC was significantly effective in increasing sugar content in sugarcane. Considering nitrate leaching and its impact on human health and the environment, NNC is potentially valuable.

Rajonee et al. prepared phosphorus and potassium compound nano-fertilizer using zeolite as a carrier material, and characterized and evaluated fertilization efficiency [77]. Material composition measured by X-ray diffraction (XRD) showed that the modified zeolite's peak position and height changed compared with unmodified zeolite, indicating that the K, P fertilizer element was successfully loaded into the zeolite and the nano-fertilizer was prepared successfully. An in vitro incubation study with the application of nano-fertilizer showed that the release of potassium was most prominent throughout the experiment and all experimental units showed the same trend to different degrees. The potassium content in the soil was maintained at a higher level with the nano-potassium fertilizer compared to control. Even though nano-fertilizer has promising performance, its large-scale implementation is limited by high cost.

Chen et al. revealed that CaSO_4_ reduced phosphorus loss from farmland through soil incubation experiments [78]. CaSO_4_ is a typical soil conditioner with strong complexation to orthophosphate. Compared to conventional crude CaSO_4_, nano calcium sulfate provides a larger surface area, higher solubility, and improved integration of fertilizer and soil, which further reduces soil phosphorus loss.

Ahanger et al. presented a nano-vermicompost organic fertilizer with particle sizes ranging from 1 µm to 200 nm after air-drying and grinding [79]. The influence of this fertilizer on the growth of tomatoes under drought stress was studied, and the results showed beneficial effects on growth, oxidative stress parameters, antioxidant, nitrogen metabolism, osmotic accumulation and mineral elements under drought stress, which demonstrated its beneficial contribution in preventing drought-mediated damage to tomatoes.

Currently, NMs in fertilizer are mostly reported from validation experiments illustrating that NMs can be used as a fertilizer directly or as auxiliary materials to indirectly improve fertilizer utilization. The lack of systematic theoretical studies on interactions and material exchange among NMs, plant root systems, root physical and microbial environments, and plant systems hinders the development and implementation of nano-fertilizer formulations.

The reported mechanisms by which NMs act as fertilizer or fertilizer synergists to affect plant growth can be summarized as the following aspects: First, reducing fertilizer particle size to nanoscale is expected to improve the solubility and dispersion potential of fertilizer nutrients. Furthermore, due to the small size effect of NMs, they have unique potential to easily pass through the membrane barriers of plant cell walls, thereby offering altered uptake kinetics of the applied nano-form fertilizers [80]. Lastly, NMs protect fertilizer by encapsulating it to reduce its volatilization, fixing it in the soil through NMs and reducing its leaching losses, thus improving its utilization and reducing environmental damage. NMs and nanotechnology in fertilizers will effectively promote fertilizers' absorption and utilization, thus improving the agronomic parameters of crops. It can also reduce the environmental pollution caused by the volatilization and leaching of chemical fertilizers.

## New applications of nanomaterials in agrochemicals

In recent years, because of the environmentally friendly control strategies advocated by Integrated Pest Management, the rapid rise of organic farming, and the increased awareness of environmental protection and food safety, many eco-friendly agrochemicals, such as bio-pesticides have received lots of attention, NMs are subsequently applied to these chemicals [81].

### Nanomaterials for bio-pesticide applications

The ecological and health threats associated with applying chemical pesticides are increasingly concerning. According to the reports of Tang and Tackenberg et al., in the past few years, bee numbers have decreased markedly and trace pesticides have been detected in 75% of the world's honey, particularly neonicotinoids, including imidacloprid, thiacloprid, and thiamethoxam. With this background, some environmentally-friendly agricultural solutions for pest control that pose little risk to humans and beneficial microorganisms have gained more attention [82, 83].

Compared with chemical pesticides, bio-pesticides are more eco-friendly, and they have become more common for pest and disease control in plants. Currently, the market share of bio-pesticides is increasing, with the following categories [84].

1. Microbial pesticides, developed using living microorganisms, specifically subdivided into bacterial pesticides, such as *Bacillus thuringiensis* (*Bt*), *B. subtilis*, and *B. cereus*; and fungal pesticides, such as *Beauveria bassiana* (*Bals*.), *Metarhizium anisopliae*, (*Paecilomyces lilacinus* (Thom.) Samson), and *Verticillium*.
2. Viral pesticides, including nuclear polyhedrosis virus (NPV), granulosa virus (GV), and genetically engineered rod-shaped virus.
3. Plant-derived pesticides are natural products in plants, some of which have insecticidal and fungicidal effects, such as EOs, bittersweet, pyrethrins, indocyanin.
4. Agricultural antibiotics are microbial secondary metabolites produced by bacteria, fungi, actinomycetes, and other microorganisms in the fermentation process, which can be used to control agricultural pests. Examples include AV, bupropion, erythromycin, and liuyangmycin.
5. Biochemical pesticides, such as vinblastine, erythromycin, and gibberellic acid.
6. Plant immunity elicitor-inducing antibacterial agents, which allow plants to fight against pests and diseases by enhancing their immune function, such as chitosan and activating proteins.

Bio-pesticides also include natural enemy pesticides, such as *Trichogramma dendrolimi* (Matsumura) and *T. chilonis* (Ishii).

However, bio-pesticides have their own drawbacks. For example, microbial pesticides and antibiotic pesticides are easily affected by external environment factors such as temperature, humidity, and light, making the AIs unstable. Using NMs as carriers is one way to solve the stability and utilization issues of bio-pesticides effectively (Table 2).

**Table 2:** Nanomaterials applied to bio-pesticide and their functions

Bio-pesticide category	Active Ingredients (AIs)	Material(s)	Function	Ref
Microbial pesticides ( Bacterial pesticide)	*Bacillus thuringiensis* (*Bt*)	Nanotubular sodium titanate	Effective in controlling cotton leaf-worm	[86]
Microbial pesticides ( fungal pesticides)	*Beauveria bassiana* (*Bals*.)	Silica nanoparticles and carbon fibers	Improving mortality to larvae of potato *Spodoptera litura (S. litura)*	[88]
Plant-derived pesticides	Essential oils	Chitosan-coated nanosilver	Synergistic effect against a wide range of microorganisms	[89]
Agricultural antibiotics	Avermectin	Poly (ethylene glycol)-carboxymethyl cellulose (PEG-CMC)	Improved the anti-UV ability & increased the biocompatibility of the Avermectin	[33]
	Validamycin and thifluzamide	Polylactic acid	The nanoparticles prepared by compounding Validamycin with chemical pesticides showed better control of rice sheath blight, which was 4.2 times more effective than the control	[91]
Biochemical pesticides	Gibberellic acid (GA)	layered double hydroxides (LDH)	Promoted plant growth	[93]
Plant immunity elicitor-inducing antibacterial agents	Chitosan & Zinc oxide	Chitosan encapsulated zinc oxide nanocomposite	As an efficient biocompatible elicitor to improve agronomic traits of crops	[95]

When *Bt* forms spores accompanied by the production of insecticidal crystal protein, which is a protein of length 130–140 kDa, it needs to be solubilized in an environment with a pH > 9.5 to be activated and then become toxic. The midgut of lepidopteran larvae is a strong alkaline environment, and when the larvae intake *Bt*, it is activated and releases 60–70 kDa of toxin, killing the target. Therefore, the smaller the particle size of *Bt*, the better its performance. Usually, top-down approaches of microionization are employed to make *Bt* in the nanoform. These approaches, including jet milling, ball milling, and high pressure homogenization, could convert the coarse powder to ultrafine powder, and are easy to scale up and give reproducible results [85]. However, the balls used in the milling may produce contamination of the final product, and heat generation during high speed milling process could reduce the viability and efficacy of *Bt*. Therefore, an increase of using NMs to load *Bt* is observed. The preparation of NMs loaded *Bt* could not only obtain ultrafine formulations, but also improve the stability and shelf life of biological pesticides in the environment. Zaki et al. studied the application of NMs in *Bt* formulations and investigated a complex of nanotubular sodium titanate and *Bt* as a novel nano-pesticide against cotton leaf-worm. The preparation was carried out by mixing the NMs sodium titanate and *Bt* in water at a mass ratio of 1:2, sonicating for 10 min, then stirring with a magnetic stirrer, and finally drying [86]. The bacterial nanocomposites showed an improvement in insecticidal activity against cotton leaf worm, *Spodoptera littoralis* and a stronger effect on the different biological features of pest than bacteria alone.

In order to address the poor stability and low utilization of *Bt*, excipients were also added to prevent settling of suspensions; preservatives were added to extend shelf life; and for powders, additives were applied to improve their wetting and spreading properties. UV shielding agents were also used to prevent rapid photolysis after spraying. These improvements resulted in a sixfold increase in the utilization of AIs [87].

*Beauveria bassiana* (*Bals*.) is a fungal microbial pesticide, usually prepared with vegetable oil and mineral oil as dispersion media to obtain oil suspensions for use. *B. bassiana* is susceptible to decomposition by UV irradiation, so Hersanti et al. used silica nanoparticles (Silica Nps.) and carbon fibers as carriers for B. bassiana to control potato *Spodoptera litura* (*S. litura*) [88]. Silica Nps. and carbon fibers can also be used as fertilizers to provide nutrients to plants while protecting them from biological stress. A total of 16 treatments (control treatments were included) were designed to assess the effect of applying different concentrations of the mixture *B. bassiana* + Silica Nps. + carbon fiber on the mortality of *S. litura.* The results showed that the application of the mixture of *B. bassiana* + Silica Nps. 5% + carbon fiber had the highest mortality rate (53.33%) on *S. litura* larvae, while the single *B. bassiana* treatment had a mortality rate of 6.67%.

Plant-derived pesticides are also important bio-pesticides, and currently, EOs and neem-derived products are the most widely used [89]. EOs, an important natural source of pesticides, are mostly lipophilic and interfere with insects' normal metabolic, biochemical, and physiological reactions, which eventually affect their behavior. NMs applied to plant EOs show a synergistic effect on EO bioactivity. Cinteza et al. mixed chitosan-coated nanosilver with EOs in specific ratios, and the physical and chemical properties and biological activity of the system remained stable after mixing. In addition, a synergistic effect from the antibacterial activity test was observed, and the mixture was effective against a wide range of microorganisms with minimal side effects [90].

NMs are applied in antibiotic bio-pesticides to raise the stability and utilization of AIs. AV is the most widely used antibiotic-based bio-pesticide. As noted earlier, NMs improved the anti-UV ability of AV, increased the biocompatibility of the AIs, and improved its utilization.

In addition, microbial pesticides and chemical pesticides can be loaded together through NMs to form a dual-functionalized system, which has a synergistic effect on the bioactivity. Cui et al. used water–oil-water double emulsion method combined with high-pressure homogenization technology to prepare PLA nanocapsules containing validamycin and thifluzamide (VTNC) for the control of rice sheath blight [91]. The VTNC shown an average diameter of 265.3 ± 0.8 nm and polydispersion index of 0.034 ± 0.030, and these spherical particles were well dispersed, smooth in surface, and equal in size. The bioactivity evaluation results of VTNC against *Rhizoctonia solani*. showed that dual-functionalized system was significantly better than that of the commercial formulations and a clear synergistic effect between the two AIs was observed.

Gibberellic acid (GA) is an important biochemical pesticide, and is relatively widely used. GA stimulates plant growth, such as promoting the formation of male flowers [92]. Hafez et al. used layered double hydroxides (LDH) as a potential carrier for a GA formulation [93]. GA@LDH nano-formulation was synthesized using the memory-effect property of LDH. TEM showed a hexagonal flake-like shape with a lateral dimension ranging from 10 to 20 nm. The GA@LDH nano-formulation promoted plant growth with a significant increase in stem length over the control at the same concentration, and also showed better bioavailability and improved safety compared to the control. This study highlights a potential nanohybrid formulation form of GA using the memory effect property of the LDH clay material.

As a plant immunity elicitor, chitosan stimulates plant growth, induces disease resistance in plants, and produces immunity and inactivation against various fungi, bacteria, and viruses [94]. Asgari-Targhi et al. synthesized chitosan/zinc oxide nanocomposite (CS-ZnONP) and evaluated their biologically in vitro conditions [95]. The nanocomposite was able to improve plant growth and biomass more than bare ZnONPs use. Plants with CS-ZnONP increased chlorophyll (51%), carotenoids (70%), proline (twofold) and protein (about twofold) concentrations than with bare ZnONPs. Adding CS-ZnONP to the seed medium increased enzymatic antioxidant biomarkers (catalase and peroxidase). The activity of phenylalanine ammonia-lyase showed a similar significant upward trend. CS-ZnONP treatment greatly enhanced the accumulation of alkaloids (60.5%) and soluble phenols (40%). Micropropagation experiments showed that CS-ZnONP treatment promoted organ development. Overall, CS-ZnONP can be considered as a highly effective biocompatibility elicitor.

The integrated system of pesticides and fertilizers constructed with NMs and nanotechnology can achieve the co-delivery of pesticides and fertilizers, which is of great potential in reducing labor cost and giving full play to the synergistic effects of fertilizers and pesticides [96].

### Nanomaterial-mediated nucleic acid pesticides system

**Fig. 4:**
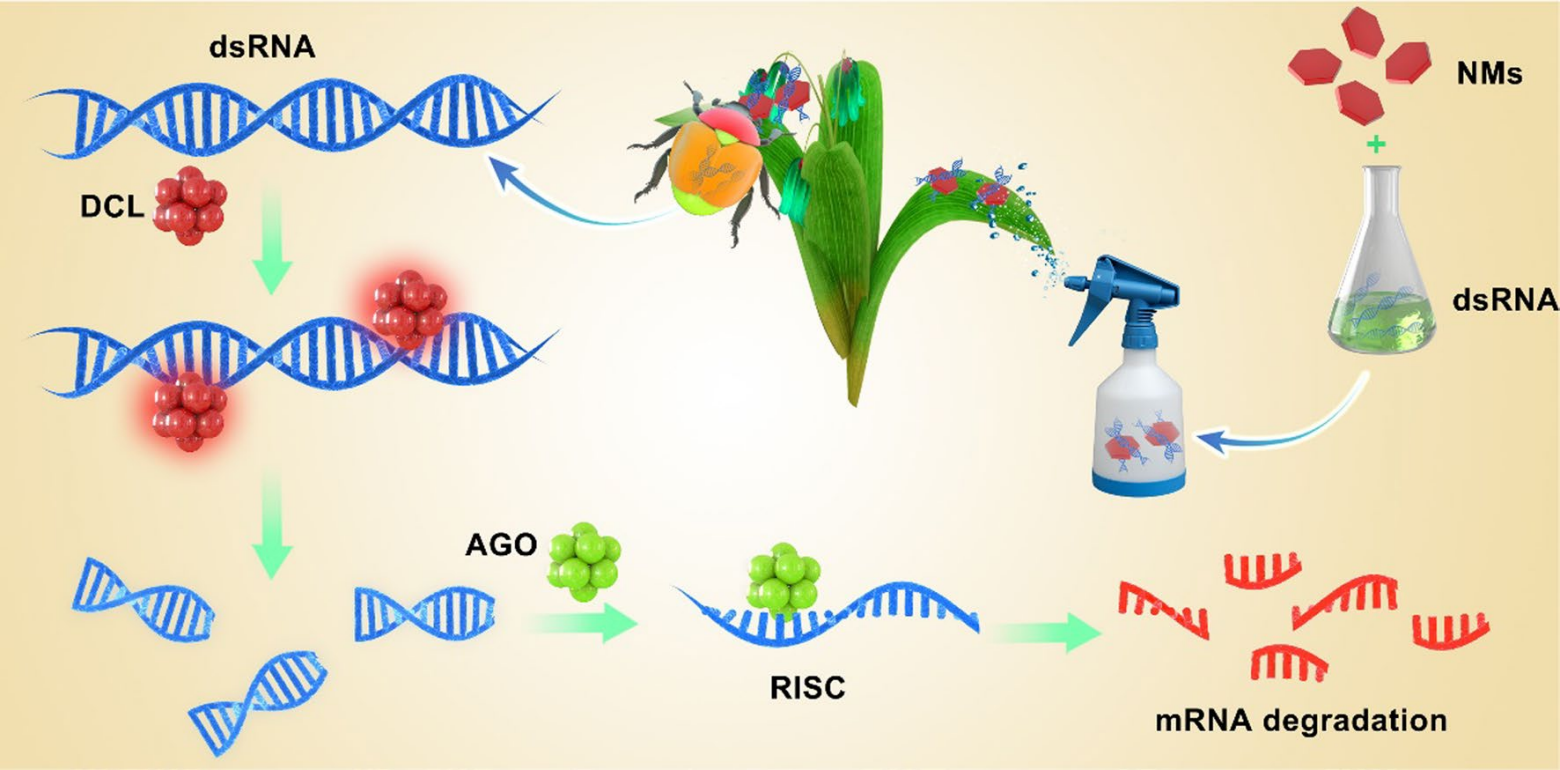
Nanomaterial-mediated nucleic acid pesticides system. NMs protect nucleic acids, reduce degradation and help them enter the cell of target pests. Then, the RNAi program is initiated to interfere with the normal transcriptional pathway of the target and achieve the effective control of the pests

Nucleic acid pesticides are based on the introduction of exogenous nucleic acid fragments into the target, which interferes with the normal transcription of the target gene, resulting in the death of the target. The more studied are RNA pesticides. RNA pesticides are used to protect crops with RNA interference (RNAi) technology. RNAi delivers double-stranded (ds)RNA /small interfering (si) RNA to insects, silencing genes and affecting normal genetic coding, ultimately killing the pests (Fig. 4.) [97]. RNA-pesticides work through the initiation, effect, and cascade amplification of RNAi by Dicer-like (DCL), Argonaute(AGO), and RNA-dependent RNA polymerase (RdRP). The RNA-induced silencing complexes (RISC) assembled by functional small RNAs and AGO enzymes are the minimal functional units of gene silencing [98]. DCL participates in cleavage and processing of small RNA precursors (dsRNA or microRNA precursors, etc.) to form mature small RNA sequences [99]. AGO is implicated in the recognition and selective binding of small RNAs to promote the formation of functional RISC complexes and mediate target sequence degradation or translational repression [100]. RdRP is mainly associated with the generation and replication of secondary small RNAs, further enhancing and amplifying the silencing effect [101]. Therefore, functional small RNAs are the core elements of biologically mediated RNAi-dependent gene silencing, and DCL, AGO, and RdRP are critical for maintaining the generation, transmission, and amplification of RNAi-mediated gene silencing signals [102].

RNA pesticides are safe and environmentally friendly because of their strong specificity, low development cost, and safety. These pesticides meet current criteria for sustainable agriculture, as a potential environmentally friendly and effective pesticide for plant protection. However, RNA pesticides are unstable, easily degraded, and decompose themselves before reaching the target. NMs are applied in RNAi delivery systems in order to protect siRNA and improve its efficiency for entering into pests [103].

Mitter et al. demonstrated the possibility of using LDH-bioclay as a carrier for transporting dsRNA applied to crop protection, as an alternative to the traditional transgenic treatment method [104]. LDH-bioclay firmly adhered to the leaf surface of *Vigna unguiculata ssp. unguiculata* (cowpea) and *Nicotiana tabacum cv.* Xanthi nc even after vigorous rinsing. Longer stability of dsRNA protected by LDH-bioclay was ensured under the environmental conditions of application, with a persistence period of up to 20 days. The sustained release of dsRNA was facilitated by the formation of carbonic acid on the leaf surface from atmospheric CO_2_ and H_2_O.

NMs also serve as DNA nanocarriers. Kwak et al. demonstrated the possibility of using single-wall chitosan-complex carbon nanotubes for chloroplast transformation [105]. They prepared single-wall carbon nanotube carriers (SWNT) based on chitosan to secure plasmid DNA (pDNA) and deliver it to the chloroplast. Chitosan-modified SWNT was rationally designed using the lipid exchange envelope penetration model to maximize the efficiency of carrying the pDNA-SWNT complex into chloroplasts. By using pDNA encoding a yellow fluorescence protein reporter gene, Kwak et al. demonstrated chloroplast-targeted gene delivery and transgene expression in mature arugula, dulse, spinach, and tobacco plants as well as isolated *Arabidopsis thaliana* protoplasts.

Demirer et al. noted that high aspect ratio carbon nanotubes enable delivery of functional genetic material without DNA integration in mature plants [106]. Therefore, this delivery system could achieve efficient genome modification without transgene integration, thus avoiding strict genetically modified organisms regulations. Moreover, this work shows that carbon nanotubes can protect DNA cargo from nuclease degradation. Such studies showed the potential of delivering biomolecules into plant cells through DNA nanostructures, and DNA nanocarriers provide important guidance for the development of effective ligand-mediated nucleic acid delivery systems.

### Nanomaterials applied in plant growth regulators

Plant growth regulators (PGRs) are a special family of pesticides mainly manufactured by artificial synthesis and from substances with high biological activity. They regulate the growth and development of plants at very low concentrations. Similar in concept to PGRs are the five major categories of endogenous plant hormones, namely auxin (IAA), gibberellins (GA), cytokinins (CTK), abscisic acid (ABA), and ethylene (ETH). PGRs and plant hormones have the same chemical structure and biological effects, only the derivation is different [107]. NMs in combination with PGRs are mainly used to help detect trace amounts of plant hormones in plants and flexibly adjust the hormone levels to obtain the best production value. Moreover, NMs assist in the absorption and transport of PGRs into the plant.

Chen successfully prepared β-Cyclodextrin modified magnetic graphene oxide material (Fe_3_O_4_@SiO_2_/GO/β-Cd) [108]. This is a novel magnetic sorbent for the enrichment and purification of PGRs in plants before GC–MS detection. The advantages of this material include its magnetic separation properties, large surface area due to its small size, high supramolecular recognition rate, low cost, and environmental friendliness. Using this material, accurate test results were obtained for trace levels of five PGRs in real vegetable samples, with good linear relationships in the range of 1–100 lg/kg with determination coefficients (R^2^) from 0.9983 to 0.9996. Similarly, Li et al. used crystalline porous polymer material to prepare a sorbent for PGR detection [109]. They chose covalent organic frameworks (COFs) to synthesize magnetic covalent organic frameworks Fe_3_O_4_@COF(TpDA), which were constructed with organic building units via covalent bonds. The results showed good linearity (R ≥ 0.9990) and low detection limits (4.68–7.51 μg/L) with a recovery of 83.0%-105.0%, suggesting that this method can be used to detect PGRs in fruits and vegetables.

Santo Pereira et al. developed alginate/chitosan (ALG/CS) and chitosan/ tripolyphosphate (CS/TPP) nanocarriers to encapsulate the PGR GA-3 [110]. ALG/CS nanoparticles with and without GA-3 had a mean size of 450 ± 10 nm, PDI of 0.3, and a zeta potential of + 27 ± 3 mV. ALG/CS-GA-3 nanoparticles with 100% encapsulation efficiency and bioactivity results showed an increase in leaf area, chlorophyll, and carotenoid content compared to the control.

NMs themselves can also regulate the growth and development of plants. Khodakovskaya et al. discovered that multi-walled carbon nanotubes (CNTs) influence plant growth and the composition of soil microorganisms [111]. CNTs were applied to tomato cultivation and there were twice as many fruits and flowers in tomatoes with CNTs than in the control. In a study of soil microbial communities, the relative abundances of *Bacteroidetes* and *Firmicutes* increased, whereas *Proteobacteria* and *Verrucomicrobia* decreased with increasing concentrations of CNTs. Chakravarty et al. used graphene quantum dots as enhanced PGRs to validate their effects on coriander and garlic growth [112]. The results showed that graphene quantum dots used to treat seeds promoted the growth rate of coriander and garlic plants, including leaves, roots, shoots, flowers and fruits. Graphene quantum dots can be used in a variety of other plants for high production.

### Nanomaterials applied in pheromones

**Fig. 5:**
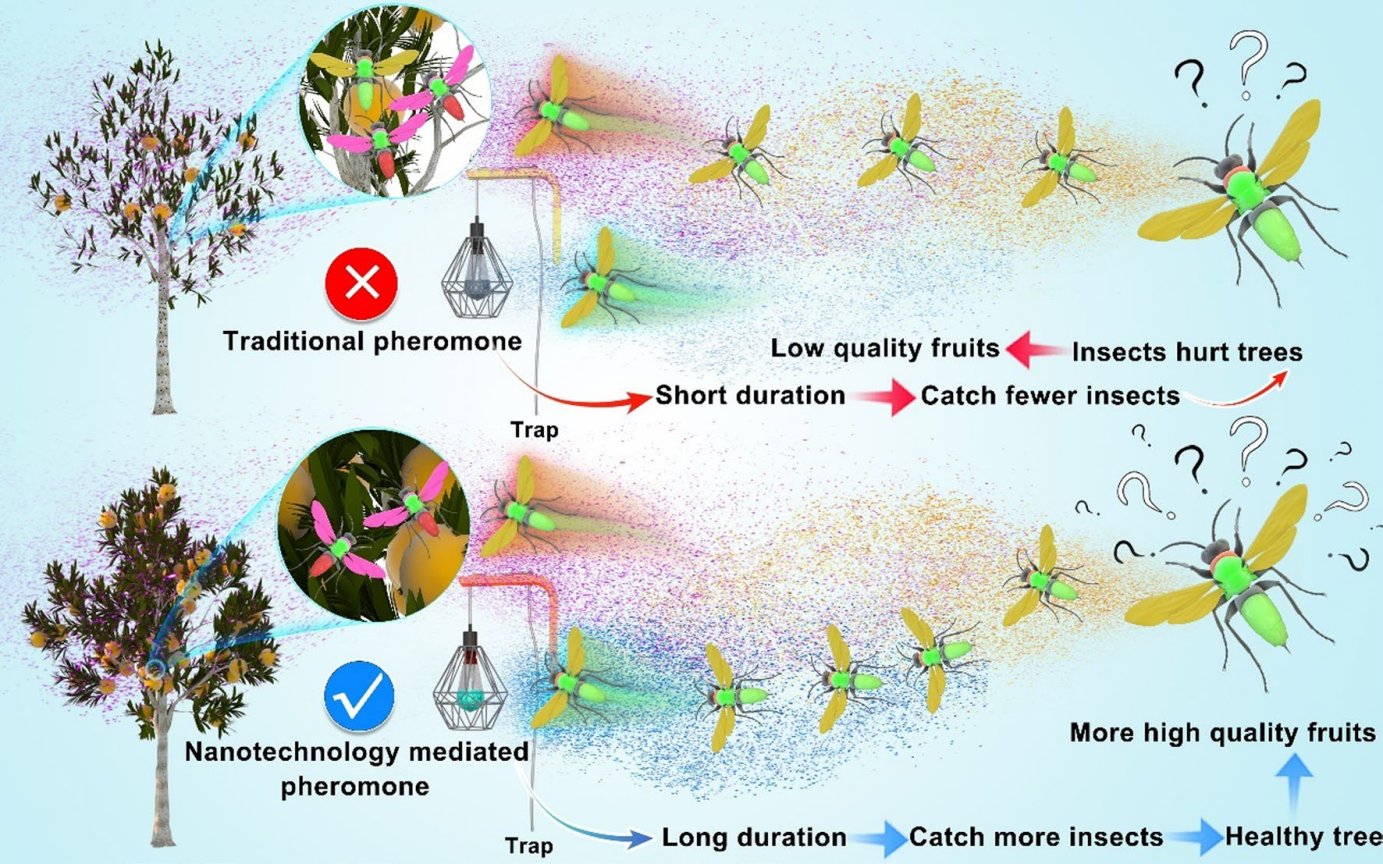
Nanomaterial loaded pheromone. Traditional pheromones products are unstable and easy to volatilize, resulting in short shelf life and unsatisfactory "Attract-and-kill" effect; Nanomaterial-loaded pheromones with environment friendly properties can realize slow-release, protect from decomposition under ambient conditions, and show excellent efficacy in the open orchard

Attract-and-kill through pheromones is one of the most potent approaches to integrated pest management [113], and continuous release of pheromone active substances is required during the pest capture period (Fig. 5.). However, due to the volatile nature of pheromones, their duration is usually very short, and frequent replacements are needed during field application. This is a major disadvantage of pheromones that needs to be solved. Larson et al. developed a controlled release polyethylene dispenser for the controlled release of pheromone AIs [114]. Seo et al. used cetyltrimethylammonium to modify A zeolite and synthesized a pheromone distributor, which has a low Si/Al ratio with nanoporous structure and possesses a high cation exchange capacity to maximize the adsorption of pheromone components [115]. Attraction test showed that the nanoporous carrier exhibited better elicitation performance than commercial product under the same environment. Correia et al. also developed a composite membrane for the slow release of pheromones using poly (butylene adipate‑co‑terephthalate) and activated charcoal, which could protect the AI from climatic factors and prolong the shelf life of the pheromone [116]. The results of diffusion experiment showed that the volatility of pheromone rhynchophorol was 100% within 24 days for the control sample, while the volatility of composite membrane-coated rhynchophorol was only 45% after 65 days.

Bhagat et al. prepared pheromone methyl eugenol (ME) nanogels using low-molecular mass gelators (LMMGs)-all-trans tri (*p*-phenylene ethynylene) bis-aldoxime [117]. The preparation was based on the self-assembly by weak intermolecular non-covalent interactions (e.g., hydrogen bonding, π-π stacking and van der Waals) of LMMGs, which leads to specific solvent gelation. The gel system was a three-dimensional nanoscale supramolecular network structure, providing high pheromone retention capacity. In addition, the nanogel was insoluble in water, which protected ME from decomposition caused by light, temperature and humidity in the environment, thus prolonged the shelf life. The perpetration did not require the use of any additives, such as crosslinkers and antioxidants, so it was an environmentally friendly agrochemical. The results of field trapping tests showed that in the luring test of *B. dorsalis*, treatment 1 (ME alone) and treatment 2 (ME in nanogel) were basically equal in the first three days, while from the 8th day, treatment 2 showed a more effective effect than treatment 1. Therefore, nanogel immobilized ME was an effective agent for the control of *B. dorsalis*.

## Future challenges in the development of nano-agrochemicals

NMs are still being explored for applications in agrochemicals, and safety assessment, production cost, evaluation standards, registration polices and public concern issues must be addressed. A critical assessment of the impact of nanoparticles is necessary before they are determined to be safe to use [118].

### Environmental safety assessment

There are concerns about the potential safety risks of NMs to non-target organisms as well as to humans. Juárez-Maldonado et al. summarized that the direct interaction of NMs with microbial cells is also quite complex and may cause specific changes in microbial cell physiology and gene expression that can lead to proliferation of any kind of cell [119]; such risks may reduce the diversity and abundance of specific microbial groups in the soil [120]. Risk assessment of NMs for agrochemicals primarily remains in the laboratory stage and the real scenarios of application do not have enough risk assessment. Various application environments may lead to variable results. For the design and predict the environmental fate of the nanoscale agrochemicals, Zhang et al. proposed to integrate existing nutrient cycling and crop productivity models with nanoinformatics approaches to optimize targeting, uptake, delivery, nutrient capture, and long-term effects on soil microbial communities to design nanoscale agrochemicals that combine the best safety and functional properties [121]. Kah et al. suggested that nano-agrochemicals should be extensively tested for effectiveness and environmental effects under field conditions [122]. Further work needs to be done to evaluate nano-agrochemicals to assess the advantages and new risks of existing products in a reasonable manner.

### Production cost, evaluation standards and registration policies

Though nano-enabled agrochemicals may offer a range of benefits, they are still in the early developmental stage. Several companies have deposited patents comprising numerous protocols for production and application of nano-products, whereas, the commercial nanotechnology-based products is still relatively limited in quantity and needs further development. According to the Nanotechnology Products Database available at Statistic Bank (StatNano), there are 9420 nanotechnology-based products are commercialized, only 229 nano-products launched for agriculture application [123]. Most of these products are nano-fertilizers (43%), and nanoformulations aimed at improving plant survival against disease, pest and other stress represent together 28% of these products. The high production costs and complexity of the production process make the use of NMs in agriculture a low-margin industry that cannot generate significant economic returns on the initial production investment [124]. This is a major constraint to the application of NMs in agrochemicals on a large scale. Furthermore, there is a lack of uniform methods and standards among government authorities relating to NMs [125]. The existing methods of government regulators are not applicable or cannot correctly evaluate the safety risks of nano-pesticides, and there is an urgent need to study and establish corresponding evaluation methods and standards, for the large-scale application of NMs in agrochemicals. The pesticide registration authorities in the US and European countries have issued registration policies for nano-pesticides. The US EPA has used the Federal Insecticide, Fungicide, and Rodenticide Act (FIFRA) to require the registration of nano-pesticides as “new” substances and to refine relevant registration policies. The EU member states register pesticides under the Plant Protection Products Regulation, which applies to the registration of single pesticides and mixtures, regardless of size, and this includes the registration of nano-pesticides [126].

The public's acceptance of new technologies also hinders the industrialization of NMs in agriculture to a certain extent, especially as agriculture is closely related to food safety and people are likely to be sensitive to the application NMs in agriculture.

## Prospects for nanomaterials applied in agrochemicals

In this review, we discussed the application of NMs and nanotechnology in plant protection, including the use of NMs in constructing nano-pesticide formulation systems, nano-fertilizers, bio-pesticides, nucleic acid pesticides, PGRs, and pheromone. We also noted the challenges of industrial production of NMs for plant protection. Although nano-agrochemical delivery is at an early stage of development, it is expected that this application will improve the efficiency of agrochemicals and reduce environment pollution. More research is required to facilitate their large-scale application. In the future, the research focus of nano-agrochemicals are as follow; (1) competition with the conventional formulations in performance, cost and scale manufacture technology, especially in the prospect of commercialization; (2) establishment of new evaluation standards of nano-agrochemicals on product quality and safety; (3) precise controlled-release systems able to response to the changes in environmental stimuli such as pH, light, temperature, enzyme activity, etc.; (4) establishment of specific using guidelines and effective large-scale field application technology of nano-agrochemicals to facilitate their extension in agriculture.

Industrial production of nano-agrochemicals prepared by NMs and nanotechnology are influenced by many factors including product economics, environmental factors, evaluation standards, using guidelines and policy issues, but the benefits of NMs and nanotechnology applied in agricultural are likely to be monumental because it can improve the efficacy, accuracy and targeting of agrochemicals. In addition, the introduction of reasonable policies and popularization of the new technology among the public are also beneficial to promote its development.

## Data Availability

Not applicable.

## Abbreviations

NMs: Nanomaterials
PGRs: Plant growth regulators
NSMs: Nanostructured materials
ENMs: Engineered nanomaterials
PGRs: Plant growth regulators
AIs: Active ingredients
WP: Wettable powder
EC: Emulsifiable concentrate
AV: Avermectin
UV: Ultraviolet
PEG-CMC: Poly (ethylene glycol)-carboxymethyl cellulose
CPF: Chlorpyrifos
PA: Attapulgite
CA: Calcium alginate
ROS: Reactive oxygen species
PLA: Polylactic acid
NaAlg: Sodium bentonite
EOs: Essential oils
TEM: Transmission electron microscopy
PD: Potato dextrose
DLS: Dynamic light scattering
XRD: X-ray diffraction
Zn: Zinc
Se: Selenium
N: Nitrogen
P: Phosphorus
K: Potassium
Ca: Calcium
*Bt*: *Bacillus thuringiensis*
*Bals*.: *Beauveria bassiana*
NPV: Nuclear polyhedrosis virus
GV: Granulosa virus
PHSNs: Porous hollow silica nanoparticles
GA: Gibberellic acid
LDH: Layered double hydroxides
PEG-CMC: Poly (ethylene glycol)-carboxymethyl cellulose
RNAi: RNA interference
(ds)RNA: Double-stranded RNA
(si)RNA: Small interfering RNA
DCL: Dicer-like
AGO: Argonaute
RdRP: RNA-dependent RNA polymerase
RISC: RNA-induced silencing complexes
SWNT: Single-wall carbon nanotube carriers
pDNA: Plasmid DNA
IAA: Auxin
GA: Gibberellins
CTK: Cytokinins
ABA: Abscisic acid
ETH: Ethylene
COFs: Covalent organic frameworks
ALG/CS: Alginate/chitosan
CS/TPP): Chitosan/ tripolyphosphate
CNTs: Carbon nanotubes
ME: Methyl eugenol
LMMGs: Low-molecular mass gelators
FIFRA: Federal insecticide, fungicide, and rodenticide act
